# The Challenges of Studying the Anaerobic Microbial World

**DOI:** 10.1264/jsme2.ME2904rh

**Published:** 2014-12

**Authors:** Koji Mori, Yoichi Kamagata

**Affiliations:** 1 NITE Biological Resource Center (NBRC), National Institute of Technology and Evaluation (NITE), 2–5–8 Kazusakamatari, Kisarazu, Chiba 292–0818, Japan; 2 Bioproduction Research Institute, National Institute of Advanced Industrial Science and Technology (AIST), 2–17–2–1 Tsukisamu-Higashi, Toyohira-ku, Sapporo, Hokkaido 062–8517, Japan

Studies on strictly anaerobic microorganisms represent one of the most challenging areas of research, because anaerobic conditions (oxygen-free) need to be reconstructed to understand microbial activities and to obtain enrichments and pure culture. It is well-known that the micro/macro anaerobic environments are present everywhere on the Earth, and anaerobes comprise complex communities that play an important role in the carbon, nitrogen, and sulfur cycles on earth (49). Microbial community studies using 16S rRNA gene, as well as various functional genes, have offered new insights into anaerobic microbial ecology. Furthermore, numerous new lines of evidence offered by recent omics-driven and high-throughput sequencing studies provide a new vision of the anaerobic microbial world.

In the current issue of Microbes and Environments, Cheng *et al.* (4) reported that different types of sulfate-reducing prokaryotes that can be grown under specific temperatures ranges were detected in sulfate-amended enrichment cultures of muddy fluids taken from a Taiwanese terrestrial hydrocarbon seep, and that indigenous microbial communities might change based on the dynamic environmental fluctuations in volcanic mud ecosystems. Sulfate-reducing prokaryotes are frequently found in sulfate-supplied environment and are capable of growing on a variety of electron donors. In gas seeps and oil fields, the presence of various sulfate-reducing prokaryotes (9, 27, 30, 39) is associated with their potential to degrade anaerobic aromatic compounds and hydrocarbons. In fact, some sulfate-reducing bacteria are known to be decomposers of these compounds (*e.g.*, *Desulfobacula toluolica*, *Desulfogloeba alkanexedens*, *Desulfosarcina* sp.) (5, 11, 12, 48). In addition, a recent study reported that the hyperthermophilic sulfate-reducing archaeon *Archaeoglobus fulgidus* oxidizes long-chain *n*-alkanes (24). Together with these findings, sulfate-reducing bacteria are also known to be an important microbial group as syntrophic partners in anaerobic ecosystems. Consortia of anaerobic methanotrophic archaea and sulfate-reducing bacteria contribute to the global methane consumption in methane-seeps (41). In addition, hydrogen and sulfur-compounds are syntrophically utilized by sulfate-reducing bacteria, sulfur-oxidizing bacteria, fermenters and anoxygenic photosynthetic bacteria in hot springs and hydrothermal fields showing the complexity and importance of synrtophic associations between organisms (10, 25, 36, 38).

Methanogens play a key role in anaerobic ecosystems, and represent the most important member for the effective organic degradation and the recovery of methane as energy in anaerobic digesters treating various types of wastewater (3, 13, 34, 50). Due to their growth under very low redox conditions, their cultivation and physiological analyses require special laboratory techniques and apparatus (8, 22). Methanogens are phylogenetically widespread among the phylum *Euryarchaeota*, and the discovery of new lineages is ongoing. *Methanomassiliicoccus luminyensis* was isolated from human feces (7) and is the first methanogenic representative belonging to the class *Thermoplasmata* (14). The class originally consisted of acidophilic and aerobic archaea (42) and of uncultured lineages retrieved from hydrothermal fields (46), rice fields (23), and so on. Isolation of *Thermoplasmata-*related methanogens within the unexpected lineage suggests that methanogens are phylogenetically more diverse than previously thought, and holds the promise of the discovery of as-yet-unrecognized methanogens (6, 35). The genus *Methanocella* also represents a novel lineage of methanogens, formerly called “Rice Cluster I”, and the only cultivated representative belonging to the order *Methanocellales* (44). Sakai *et al.* successfully isolated *Methanocella paludicola* using an elaborate enrichment method: low-hydrogen conditions were created by using *Syntrophobacter fumaroxidans* as a hydrogen-producing fermenter (43) so that methanogens that favor low concentrations of hydrogen was selectively enriched and isolated. This example makes it clear that inventive approaches to cultivation provide opportunities for success. On the other hand, it is also important to easily and efficiently create the conditions for cultivation of fastidious anaerobic microorganisms like methanogens. Carbonero *et al.* (2) reported that improving the culture medium made the colony formation of *Methanosaeta* species successful. Nakamura *et al.* (32) developed a simple technique for the cultivation of anaerobic microorganisms, by using a six-well plate and anaerobic gas-pack system. Subsequently, *Methanothermobacter tenebrarum* was successfully isolated using this technique (33). Clearly, increase in colony forming efficiency would facilitate not only isolation of yet-to-be cultured methanogens but further studies using genetic manipulations.

Anaerobic ammonium oxidation (anammox) is a microbial process in which ammonium is anaerobically oxidized to nitrogen gas with nitrite as an electron acceptor. Strous *et al.* first reported that this phenomenon occurs with anammox bacteria belonging to the order “*Brocadiales*” in the phylum *Planctomycetes* (45). As this process does not require a supply of oxygen or of organic substrates for stimulation of denitrification, it is expected to serve as an alternative to conventional processes used to remove nitrogen from ammonia-rich wastewater. In nature, anammox has been detected in marine and fresh-water sediments, soils, and so on (1, 52), and it is likely that anammox bacteria significantly contribute to the global nitrogen cycle (15, 16, 28). Based on enrichment studies (26, 37, 51), five candidate genera, “*Candidatus* Anammoxoglobus”, “*Candidatus* Brocadia”, “*Candidatus* Jettenia”, “*Candidatus* Kuenenia”, and “*Candidatus* Scalindua”, have been proposed (17), but none of the pure cultures have been so far isolated. Oshiki *et al.* (37) reported that two dominant enrichments, those of “*Candidatus* Brocadia sinica” and of “*Candidatus* Scalindua sp.”, were obtained by using membrane bioreactors. The fluorescence *in situ* hybridization study indicated that anammox bacteria dominated the biomass, as they accounted for more than 90% of its total biomass. Additional ecophysiological and biochemical studies using this dominated and stable enrichment are required to obtain pure anammox bacteria and to fully clarify the anammox process.

As the current issue of Microbes and Environments introduces the ecophysiological functions of anaerobes. For example, the intestinal colonization by *Lachnospiraceae* bacterial strain AJ11941 may contribute to the development of metabolic dysfunctions in obese mice (20). The gut environments may represent interesting anaerobic ecosystems to study in association with their hosts (40, 47). Cross-interactions between aerobic and anaerobic microorganisms are important factors with respect to organic matter degradation and material cycles of various types (19, 29, 31). Recent studies reported that anaerobic microorganisms use filaments (flagella and pili) for their communication and respiration, and that they are important functional parts than previously thought (18, 21). Interspecies electron transfer using conductive flagella or inorganic materials will become one of the most intriguing topics in microbial ecology.
